# Retention of the rural allied health workforce in New South Wales: a comparison of public and private practitioners

**DOI:** 10.1186/1472-6963-13-32

**Published:** 2013-01-27

**Authors:** Sheila Keane, Michelle Lincoln, Margaret Rolfe, Tony Smith

**Affiliations:** 1 University Centre for Rural Health, University of Sydney, PO Box 3074, Lismore, New South Wales, Australia; 2 Faculty of Health Sciences, The University of Sydney, Lidcombe, New South Wales, Australia; 3 Southern Cross University, Lismore, New South Wales, Australia; 4 University of Newcastle Department of Rural Health, Tamworth, New South Wales, Australia

## Abstract

**Background:**

Policy initiatives to improve retention of the rural health workforce have relied primarily on evidence for rural doctors, most of whom practice under a private business model. Much of the literature for rural allied health (AH) workforce focuses on the public sector. The AH professions are diverse, with mixed public, private or combined practice settings. This study explores sector differences in factors affecting retention of rural AH professionals.

**Methods:**

This study compared respondents from the 2008 Rural Allied Health Workforce (RAHW) survey recruiting all AH professionals in rural New South Wales. Comparisons between public (n = 833) and private (n = 756) groups were undertaken using Chi square analysis to measure association for demographics, job satisfaction and intention to leave. The final section of the RAHW survey comprised 33 questions relating to retention. A factor analysis was conducted for each cohort. Factor reliability was assessed and retained factors were included in a binary logistic regression analysis for each cohort predicting intention to leave.

**Results:**

Six factors were identified: professional isolation, participation in community, clinical demand, taking time away from work, resources and ‘specialist generalist’ work. Factors differed slightly between groups. A seventh factor (management) was present only in the public cohort. Gender was not a significant predictor of intention to leave. Age group was the strongest predictor of intention to leave with younger and older groups being significantly more likely to leave than middle aged.

In univariate logistic analysis (after adjusting for age group), the ability to get away from work did not predict intention to leave in either group. In multivariate analysis, high clinical demand predicted intention to leave in both the public (OR = 1.40, 95% CI = 1.08, 1.83) and private (OR = 1.61, 95% CI = 1.15, 2.25) cohorts. Professional isolation (OR = 1.39. 95% CI = 1.11, 1.75) and Participation in community (OR = 1.57, 95% CI = 1.13, 2.19) also contributed to the model in the public cohort.

**Conclusions:**

This paper demonstrates differences between those working in public versus private sectors and suggests that effectiveness of policy initiatives may be improved through better targeting.

## Background

Rural health workforce shortage is an international problem which has received considerable attention in recent years [1]. Health workforce research in the medical profession is well established [2,3] while recruitment and retention of rural nurses is relatively less investigated [4-6]. Still fewer studies have focused on the allied health professions [7-9]. Retention of the existing rural allied health workforce is also problematic, with a recent study reporting that 42% of survey respondents in NSW intended to leave their job within the next 5 years [10]. Clinical consequences notwithstanding, workforce shortages can result in decreased productivity and burnout of remaining staff, as well as high vacancy and recruitment costs [11].

The development of rural health workforce policy has relied primarily on the evidence for the successful recruitment and retention of rural doctors, most of whom practice under a business model underpinned by government and private insurance rebates, as well as direct payment for services. In contrast most nurses operate as employees within public health systems. The distinctions between models of service may have consequences in developing effective retention programs. For example, the literature on retention of rural doctors does not highlight management practices as an issue, yet this is a major influence on retention of rural nurses [12].

The Rural Allied Health Workforce (RAHW) study commenced in 2008 with a survey of all allied health professionals in rural NSW, followed by a series of focus groups to add depth to survey results and inform subsequent survey analysis. The overall aim of the RAHW study was to identify factors contributing to the recruitment and retention of rural allied health professionals.

In addition to collecting demographic, education and employment characteristics, the RAHW survey contained a final section with 33 items exploring a range of relevant issues based a review of the literature [13]. Topics included locum support [14], resources [15], professional isolation [16], work roles [17], career progression [18], workload [19], access to continuing professional development [20], salary [21], management [22] and social interaction [23,24].

Much of the existing literature for rural allied health workforce focuses on retention in the public sector [25]. However, the allied health professions are diverse, with some professions working primarily in public health settings (e.g. Occupational Therapists), others primarily in private practice (e.g. Optometrists), and some that work in either sector (e.g. Physiotherapists) [10].

This study aims to explore conceptual themes represented as clusters in the 33 RAHW survey items to identify whether the issues affecting retention of rural allied health professionals differ in relation to their business model, in either the private or public sector.

## Methods

The aforementioned RAHW survey recruited from 21 eligible allied health professions across rural NSW with a sample size of 1823 respondents. Sampling rates have been previously reported [10] and were estimated against 2006 Australian Bureau of Statistics census data, except for professions not included in the census. Sampling rates ranged by profession between 30% and 54%. This project was approved by the University of Sydney Human Research Ethics Committee.

The present statistical analysis considered respondents from the public and private work sectors. Therefore, 135 RAHW respondents who worked in both the public and private sectors were excluded from analysis, as well as a further 99 respondents working in other settings such as in non-profit organisations or the military. A total of 1589 respondents were included in the comparative analysis; 833 public and 756 private.

Comparisons between groups were undertaken using Chi square analysis to measure association for age, gender, marital status, dependents, rural origin, job satisfaction and intention to leave. Age was grouped by decades from 20 to greater than 60, with 40 year olds being the comparison reference group. The Likert scaled job satisfaction variable was dichotomized into Satisfied (very satisfied or satisfied) and Dissatisfied (neutral, dissatisfied or very dissatisfied). Similarly, Intention to Leave timeframes were combined to form the dichotomous variable Intention to Leave (2 to 5 years) or Stay (10 or more years).

The final section of the RAHW survey comprised 33 questions relating to a topic relevant to recruitment and retention. Responses were measured on a five-point Likert scale from 1 = Strongly Agree to 5 = Strongly Disagree. A sixth category (N/A) was considered missing for purposes of analysis. Eight questions were excluded from the factor analysis due to missing more than15% missing values or having high correlation with other items similar in concept. A list of survey items and treatment of missing data are described in Additional file 1: Appendix A).

Factor analysis in the public sample was conducted using the remaining 25 variables while a further 3 variables were excluded from factor analysis in the private group as there were more than 30% missing values in each variable for this cohort. Missing data for included variables were treated differently in the two groups in order to maximize sample size. No variable in the public cohort was missing greater than 3.1% of responses so means were substituted for missing values, preserving 829 public records for analysis. Missing rates ranged from 2.2% to 13% in the private cohort, so mean substitution was not appropriate. Instead missing data were excluded listwise, yielding an effective sample size of 432 records for the private factor analysis.

A principle components extraction method with Varimax rotation was used, restricting factor extraction to an Eigenvalue greater than one. Reliability was assessed, accepting Cronbach’s alpha greater than 0.5 for exploratory purposes [26]. A factor value was calculated based on the mean of the contributing variables, using reverse coding to ensure that all items were ranked in the same direction.

Retained factors were included in a binary logistic regression analysis for each cohort (public/ private) predicting intention to leave. Statistical models were developed by cohort, with all univariate logistic analyses being adjusted for age group, as this is a known factor predicting intention to leave [27]. Factors that were not significant in either cohort using univariate logistic modelling were excluded from the multivariate analysis. All statistical analysis was done at 95% level of significance using SPSS version 20.

## Results

Private sector workers (n = 756) appeared to be more embedded in rural life with 627 (83%) being married compared with 616 of 833 (74%) in the public group (Chi sq = 19.3, df = 1, p < .001) and 415 (56%) having dependents as compared with 404 (49%) in the public cohort (Chi sq = 8.1, df = 1, p = .005). The groups did not differ in rural origin, with about 60% of respondents growing up in a rural area (Chi sq = 0.5, df = 1, p = .477).

There were significantly more females working in the public sector (668 public females (61%), 421 private females (39%), Chi sq = 117.7, df = 1, p < .001). However, there was no difference between genders in relation to intention to leave (174 of all males intending to leave (38%), 279 of all females intending to leave (42%), Chi sq = 1.6, df = 1, p = .211). Age group distributions differed significantly between cohorts with 14% of private group being older than 60 years compared with 4% in the public cohort (Chi sq = 82.3, df = 4, p < .001). There were also proportionately more 20–30 year olds in the public (22%) compared with private (11%) group (Chi sq = 82.3, df = 1, p < .001).

Mean ages differed by profession, ranging from 32.7 years (speech pathologists) to 52.5 years (pharmacists). The age and gender distribution of allied health professionals across the public and private sectors is shown in Table 1.

**Table 1:** RAHW survey respondents by work sector, age and gender

		**PUBLIC (N = 833)**			**PRIVATE (N = 756)**		
	**N total**	**Mean Age (yrs)**	**N Public**	**% Female**	**Mean Age (yrs)**	**N Private**	**% Female**
Audiologist	9	49.8	4	100%	49.2	5	80%
Chiropractor	81	32	1	0%	46.0	78	32%
Dietician	55	33.0	53	96%	40	1	100%
Medical Radiation Science	231	44.4	139	67%	41.0	87	67%
Occupational Therapist	135	36.4	114	97%	38.0	16	75%
Optometrist	71	n/a	0	n/a	44.5	69	38%
Osteopath	21	n/a	0	n/a	44.1	12	52%
Pharmacist	244	48.1	37	65%	55.3	199	51%
Physiotherapist	338	43.7	165	83%	46.0	166	71%
Podiatrist	52	42.6	8	88%	40.9	44	73%
Psychologist	112	45.2	80	69%	52.1	29	59%
Social Worker	103	44.5	95	84%	47.8	4	75%
Speech Pathologist	69	32.7	67	97%	31	1	100%
Other	68	47.8	49	84%	46.7	19	58%
Total	1589	41.9	833	80%	47.0	756	56%

Public respondents (87%) were nearly twice as likely to be satisfied with their job as compared with private practitioners (74%) (OR = 1.93, 95% CI = 1.43, 2.62) yet significantly more public sector respondents (47%) intended to leave their job in the next 5 years compared with the private group (35%) (OR = 1.32, 95% CI = 1.18, 1.48). The public professionals most likely to be retained were radiographers and sonographers, while private chiropractors had the highest proportion intending to stay. Those most likely to intend leaving in 2 to 5 years were psychologists (public) and pharmacists (private).

### Factor analysis

Factor analysis resulted in a 6 factor solution accounting for 56.4% of the total variance for the private group, while 7 factors were identified in the public group accounting for 52.7% of the total variance. The rotated component and correlation matrices of factor analysis for each cohort are shown in Additional file 2: Factor analysis results.

Items retained in each factor represented a common underlying construct. Factor 1 (Professional Isolation) and Factor 5 (Resources) were self-evident but the items in Factor 2 (Participation in Community) were more diverse. Factor 2 had two underlying constructs (relationships and altruism) which together inform participation in one’s community. Factor 3 (Clinical Demand) included the item on salary satisfaction, which reflected the trade off between income and workload. Factor 4 (Able to Get Away) reflected the ability to take time away from work, including the autonomy to modify work hours. The expanded “generalist” role of rural practice, combined with self-perceived expertise, underpinned Factor 6 (Specialist Generalist). Management involvement was implied in the processes of recruitment and resource allocation, and was specifically identified in the third item clustering to Factor 7 (Management). This factor was relevant only to the public sector as its contributing variables were excluded from private cohort analysis due to more than 30% missing data in that group.

**Figure 1:**
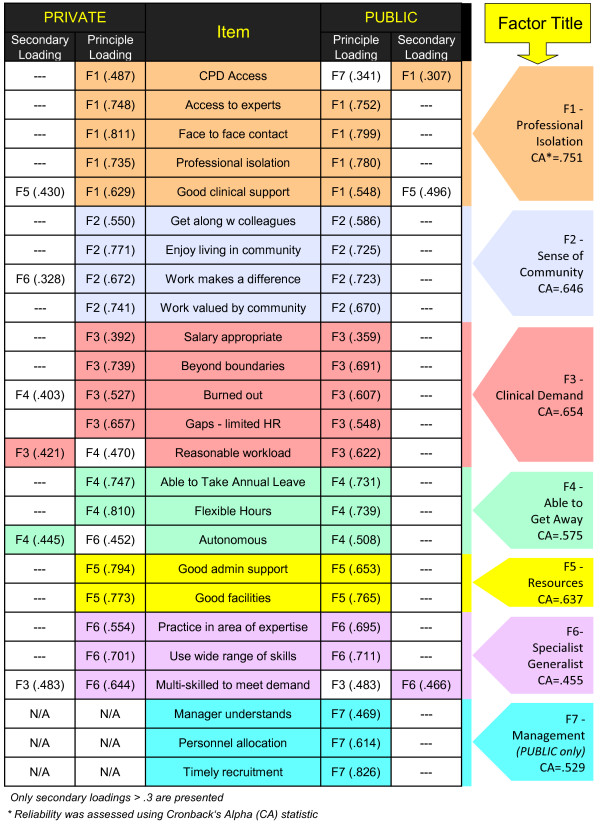
RAHW survey item loadings on factor analysis.

Results of the factor analysis differed slightly between groups. In the public sector, the item on access to continuing professional development (CPD) weighted equivalently to F1 (Professional Isolation) and F7 (Management) but was retained in F1 to be consistent with the private cohort results to enable group comparisons. With this anomaly noted, F1 (Professional Isolation), F2 (Participation in Community), and F5 (Resources) were identical in both cohorts. However, survey items distributed differently by cohort with F3 (Clinical Demand), F4 (Able to Get Away), and F6 (Specialist Generalist). In each case, the factors had a secondary loading that matched the other group so it was possible to assign variables to common factors for both groups (Figure 1). Factor 6 (Specialist Generalist) was not retained due to low reliability (α = .455).

### Regression analysis

Age group univariate logistic regression results for the private cohort showed that, compared with 40–50 year olds, AH professionals in their 20’s were about 4 times more likely to intend leaving their job and AH professionals in their 60’s were more than 11 times more likely to intend leaving their job. There was no statistical difference between 30–40, 40–50 and 50–60 year old age groups with respect to intention to leave (Table 2). This pattern was similar in the public cohort except that there was also an increased risk of 30–40 year olds intending to leave (OR 1.9, CI 1.2, 3.0).

**Table 2:** Univariate logistic regression analysis of age groups by intention to leave

		**Odds Ratio**	**95% CI**	**Sig.**	**N**
**PUBLIC** (N = 833)	20–30 years	3.72	2.43 – 5.69	.000	185
	30–40 years	1.88	1.26 – 2.83	.002	211
	50–60 years	1.50	.99 – 2.27	.055	198
	>60 years	35.41	8.22 – 152.51	.000	34
**PRIVATE** (N = 756)	20–30 years	3.38	1.98 – 5.77	.000	81
	30–40 years	.93	.57 – 1.52	.778	157
	50–60 years	1.29	.83 – 2.02	.259	185
	>60 years	7.38	4.37 – 12.46	.000	104

Age Group reference category was 40–50 years: N = 196 public, N = 215 private.

Results of univariate logistic regression analysis, adjusted for age group, are presented in Table 3. Professional isolation (F1) was a significant predictor of intention to leave in the public but not the private group. Being able to get away from work (F4) did not predict intention to leave in either group, and this factor was removed from subsequent analysis.

**Table 3:** Univariate logistic regression analysis of factors by intention to leave

		**Odds Ratio**	**95% CI**	**Sig.**	**N**
**PUBLIC** (Total n = 833)	F1 Professional Isolation (r)*	**1.53**	1.27 – 1.84	.000	784
	F2 Sense of Community*	**1.56**	1.16 – 2.10	.003	796
	F3 High clinical demand (r)*	**1.70**	1.37 – 2.10	.000	798
	F4 Get away	1.11	.92 – 1.34	.268	791
	F5 Resources*	**1.27**	1.10 – 1.46	.001	805
	F7 Management (PUBLIC)*	**1.47**	1.23 – 1.77	.000	739
**PRIVATE** (Total n = 756)	F1 Professional Isolation (r)	1.03	.84 – 1.27	.760	619
	F2 Sense of Community*	**1.53**	1.05 – 2.25	.028	642
	F3 High clinical demand (r)*	**1.48**	1.13 – 1.92	.004	568
	F4 Get Away	1.17	.96 – 1.41	.113	692
	F5 Resources*	**1.24**	1.00 – 1.53	.050	665

Analysis was adjusted for age group.

* p < .05.

In multivariate analysis, F3 (Clinical Demand) remained significant in both cohorts and F5 (Resources) was not a predictor of intention to leave in either cohort. F1 (Professional Isolation) and F2 (Participation in Community) contributed significantly to the model only in the public group (Table 4).

**Table 4:** Multivariate logistic regression analysis by intention to leave

		**Odds Ratio**	**95% CI**		**Sig.**
**PUBLIC** N = 681/833 (82%)	Age Group (20–30 years)*	**4.02**	2.47 – 6	.53	.000
	Age Group (30–40 years)*	**1.88**	1.20 – 2	.95	.006
	Age Group (50–60 years)	1.34	.85 – 2	.21	.203
	Age Group (>60 years)*	**60.42**	7.82 – 4	66.95	.000
	F1 Professional Isolation (r)*	**1.39**	1.11 – 1	.75	.004
	F2 Sense of Community*	**1.57**	1.13 – 2	.19	.008
	F3 High clinical demand (r)*	**1.40**	1.08 – 1	.83	.012
	F5 Resources	.99	.83 – 1	.18	.898
	F7 Management (PUBLIC)	1.17	.94 – 1	.46	.159
**PRIVATE** N = 461/756 (61%)	Age Group (20–30 years)*	**3.99**	2.08 – 7	.65	.000
	Age Group (30–40 years)	.82	.44 – 1	.53	.539
	Age Group (50–60 years)	1.24	.69 – 2	.22	.466
	Age Group (>60 years)*	**10.06**	4.60 – 2	2.02	.000
	F1 Professional Isolation (r)	.83	.62 – 1	.12	.203
	F2 Sense of Community	1.55	.97 – 2	.49	.068
	F3 High clinical demand (r)*	**1.61**	1.15 – 2	.25	.005
	F5 Resources	1.05	.79 – 1	.40	.717

Age Group reference category was 40–50 years.

4.2% of the public cohort and 14.2% of the private group were in the >60 age group.

*p < .05.

## Discussion

Our data show different profiles between private and public cohorts, as well as by health discipline. This diversity presents challenges for health workforce planning, suggesting that differential planning at least by sector at the federal or jurisdictional level may improve effectiveness of rural retention policy initiatives. Interestingly, gender was not a significant predictor of intention to leave, but age group did predict leaving in the 20 and 60 year old age groups. The high proportion of young practitioners in the public sector is concerning, and mentoring and rural career opportunities for this age group may improve retention in the public sector [28]. Being a significantly older group, the exodus of private practitioners may be more related to aging and retirement.

Survey items clustering as factors differed slightly between groups. For example the item on autonomy loaded more strongly to Factor 6 (Specialist Generalist) in the private group as compared with the ability to get away from work (Factor 4) in the public respondents. This difference could reflect a greater clinical autonomy in the private sector where practitioners have more control of their caseload and type of work, being limited only by their business model. There is also less bureaucracy in the private sector, permitting more flexible work practices. Autonomy and less bureaucracy in private practice settings have been associated with increased job satisfaction [29]. This advantage may be counter balanced by a greater demand, as factor analysis suggested that the ability to get away was impeded by workload in the private group but not the public cohort.

The items in Factor 2 (Participation in Community) were diverse and the underlying concept is perhaps debatable. It is interesting that items statistically clustering in this factor reflected both the quality of relationships and altruistic motivation. It has been suggested that community is formed in a context of relationships combined with purpose [30], hence the choice of title for this factor. Our results point to a desire to meet community need [31] particularly in the public cohort where Factor 2 (Participation in Community) was the strongest predictor of retention after adjusting for age group. These findings are consistent with O’Toole et al., (2008) suggesting the importance of social relationships in rural workforce retention in allied health [24] and also with research in respect of rural nurses [32] and doctors [27].

Rural AH professionals with a strong motivation to ‘make a difference’ may need to be equipped with skills to be able to manage the emotional stress of being unable to meet clinical demand [33] by use of a thorough orientation on arrival and strong mentorship for those who are geographically isolated or new to rural practice [28,34]. At a policy level, funding of rural mentorship programs seems likely to meet with as much success in allied health [35] as it has in medicine [27] and these programs could be extended to AH professionals in both public and private sectors.

Public health funding levels that do not enable sufficient allied health workforce to meet service expectations risk further pressure driving AH professionals out of rural practice. While use of qualified allied health assistants has potential to extend rural AH workforce capacity and partially alleviate workload pressures [36] this will also require negotiation of appropriate wage structures and careful regulatory policy that balances training, access and safety [37,38]. Other strategies such as a shift to a primary health care approach may ultimately reduce clinical demand, however it is likely that CPD and appropriate policy development would be required to facilitate this shift in practice.

In this study high clinical demand increased intention to leave in both the public and private cohorts, even after adjusting for all other factors. Results of RAHW focus group research [39] support this finding. The inability to meet high clinical demand has been associated with emotional exhaustion in remote area nurses [40]. Lenthall et al., (2009) suggest that this situation is “exacerbated by a low-resource environment” [41] but results of this study did not identify resources as a significant contributor to retention for either allied health cohort.

The broad nature of rural practice has consistently been identified as having a favourable influence on both recruitment and retention in rural practice [39,42,43]. Health service funding policy that rewards extended scope of practice may provide incentive to remain in rural private practice while simultaneously meeting the broad array of clinical needs in rural areas. “Rural specialist” credentialing has been suggested for rural doctors [44] and the concept could be extended to the allied health professions as a means of ensuring public safety with extended practice roles. Even within professional boundaries, rural AH professionals may lack confidence to cope with the role expansions required for rural practice [8,17]. Better access to continuing professional development (CPD) may remedy this.

The role of CPD access in rural AH workforce retention has been a matter of debate in the literature. Studies using a qualitative methodology have found that lack of CPD access could trigger a decision to leave [20,39] whereas others using survey methodology have found that CPD access improved job satisfaction but did not affect intention to leave [16,45]. Most authors agree that CPD access is just one of many factors affecting retention [4,7,18,43]. The potential of improved information technology to deliver CPD, reduce isolation and improve retention requires further research.

Because of the use of factor analysis methodology, the inability to discriminate between the CPD item and other questions on the RAHW survey was a limitation of this study. The CPD survey question loaded to F1 (Isolation) in both cohorts, but also correlated with the F7 (Management) in the public cohort. Thus it is unclear from these results whether CPD access was an influence on retention on its own, or whether it was more related to isolation or perceived management support. Improving access to CPD has been previously suggested as a retention strategy [15,20,46] and may be particularly effective where face to face meetings occur on a local or regional basis to simultaneously address isolation and training needs [39]. Perceived linkage between CPD access and management support [39] may explain why the Isolation factor was significant in the public but not in the private group on multivariate logistic regression analysis.

Poor reliability of Factor 6 (Specialist Generalist) may have been a reflection of questionnaire design as generalist work has been previously identified as favouring job satisfaction of rural AH professionals [43]. Similarly, the ability to look into the important role of locums [9] was hampered by large amounts of missing data where survey questions did not apply to the respondents’ circumstances. With several items being not applicable to private practitioners it is also likely that the questionnaire design was biased towards issues affecting the public sector. Further research is needed to clarify issues specific to the private sector in allied health.

## Conclusions

Most rural allied health workforce research has treated the group as a cohesive whole, both by profession and by sector. This paper demonstrates differences between those working in public versus private sectors and suggests that effectiveness of policy initiatives may be improved through better targeting. While orientation and mentoring are appropriate to both cohorts, early career opportunities and access to CPD may be best utilised in the public sector with a younger age demographic. In contrast locum support and recognition of “specialist generalist” expertise in more experienced rural practitioners may be more important for the older private sector. Research is needed to identify the interplay of work roles, scope of practice and access to education enabling safe and competent clinical care, and evaluation research is needed to assess the feasibility and usefulness of extending workforce through use of qualified and supervised AH Assistants to ameliorate high workloads.

## Competing interests

The authors declare they have no competing interests.

## Authors’ contributions

SK, TS and ML jointly developed the questionnaire. SK conducted the survey and Chi square analysis. MR and SK completed and interpreted the factor analysis and logistic regression. SK conducted the literature review and wrote a first draft of the manuscript. All four authors revised and approved the manuscript.

## Pre-publication history

The pre-publication history for this paper can be accessed here:

http://www.biomedcentral.com/1472-6963/13/32/prepub

## Supplementary Material

Additional file 1 **Appendix A.** List of survey items and treatment of missing data. Click here for file

Additional file 2 **Factor analysis results.** Data tables of rotated component matrices and correlation matrices. Click here for file
