# Supplementary material for: Retention of the rural allied health workforce in New South Wales: a comparison of public and private practitioners
Source: BMC Health Serv Res. 2013 Jan 27;13:32. doi: 10.1186/1472-6963-13-32 (PMC3599445; doi:10.1186/1472-6963-13-32)
Supplement: Additional file 1 — Appendix A. List of survey items and treatment of missing data. [file 1472-6963-13-32-S1.doc]

**Additional File 1: Appendix A**

**List of survey items and treatment of missing data**

| **Survey Statements Included** | % Missing  (PUBLIC) | % Missing  (PRIVATE) |
| --- | --- | --- |
| Your work hours are flexible | 2.6 | 4.1 |
| You are always able to schedule annual leave when you want it | 2.6 | 4.4 |
| You have good facilities and equipment to work with | 1.7 | 2.5 |
| You have good admin. support for enquiries, appointments, etc. | 2.3 | 11.0 |
| You have good clinical support (colleagues or therapy assistants) | 3.0 | 10.6 |
| You are working in your area of clinical expertise | 2.4 | 5.2 |
| You use a wide range of clinical skills in your work | 2.3 | 4.8 |
| You are satisfied with your access to CPD opportunities | 1.6 | 4.0 |
| You have regular face-to-face contact with colleagues in your field | 2.3 | 3.4 |
| You have good access to more experienced staff in your field | 1.7 | 9.1 |
| You feel professionally isolated | 2.3 | 4.4 |
| You have had to become multi-skilled to meet clinical demands | 2.0 | 4.8 |
| There are service gaps because of limited human resources | 1.8 | 13.0 |
| You sometimes work beyond the boundaries of your profession | 2.3 | 6.0 |
| Your workload is reasonable | 1.1 | 2.9 |
| You are autonomous and can decide your own work priorities | 1.4 | 3.2 |
| You feel ‘burned out’ | 1.7 | 4.2 |
| Your grading and salary are appropriate for the job you do | 1.3 | 13.4 |
| You get along well with your work colleagues | 1.3 | 12.8 |
| You feel that your work makes a difference to patients / clients | 1.3 | 3.4 |
| You enjoy living in your local community | 2.4 | 2.2 |
| You feel that your work is valued by the local community | 2.3 | 2.6 |

| **Survey Statements retained for PUBLIC (only) factor analysis** | % Missing  (PUBLIC) | % Missing  (PRIVATE) |
| --- | --- | --- |
| Recruitment for vacant positions always occurs in a timely way | 4.9 | 32.4 |
| Personnel are allocated according to areas of clinical need | 8.6 | 43.1 |
| You believe your manager understands your professional role | 1.3 | 46.2 |

| **Survey Statements Excluded From All Analysis** | % Missing  (PUBLIC) | % Missing  (PRIVATE) |
| --- | --- | --- |
| Locums are always available for unfilled positions | 8.6 | 17.5 |
| There is a high level of staff turnover where you work | 3.0 | 18.5 |
| You work as a member of a multidisciplinary team | 3.6 | 25.7 |
| Temporary and / or part time positions are often hard to fill | 5.8 | 28.6 |
| Locum backfill is always available when you are away on leave | 6.4 | 25.9 |
| You participate in clinical rotations / rosters across practice areas | 27.0 | 55.8 |
| Your department / practice is chronically short-staffed | 4.0 | 19.6 |
| You believe your manager values the work you do | 1.2 | 45.6 |
