# Supplementary material for: Retention of the rural allied health workforce in New South Wales: a comparison of public and private practitioners
Source: BMC Health Serv Res. 2013 Jan 27;13:32. doi: 10.1186/1472-6963-13-32 (PMC3599445; doi:10.1186/1472-6963-13-32)
Supplement: Additional file 2 — Factor analysis results. Data tables of rotated component matrices and correlation matrices. [file 1472-6963-13-32-S2.doc]

**Additional File 2: Factor analysis results**

**Rotated Component Matrix - Public Cohort**

| **Rotated Component Matrix^a^** | | | | | | | |
| --- | --- | --- | --- | --- | --- | --- | --- |
| **PUBLIC** (N=829) | Component | | | | | | |
|  | 1 | 2 | 3 | 4 | 5 | 6 | 7 |
| Face to face contact | .799 | -.037 | -.011 | .002 | -.030 | .010 | .039 |
| Professional isolation | -.780 | .196 | -.076 | -.056 | -.109 | -.100 | .022 |
| Access to experts | .752 | -.024 | -.036 | .078 | .076 | .159 | .003 |
| Good clinical support | .548 | -.049 | .079 | -.011 | .496 | .091 | .052 |
| Beyond boundaries | -.040 | .691 | .061 | .044 | .053 | -.030 | .050 |
| Reasonable workload | .069 | -.622 | -.027 | .184 | .239 | .160 | .155 |
| Burned out | -.235 | .607 | -.130 | -.237 | -.035 | -.101 | -.173 |
| Gaps - limited HR | .002 | .548 | .094 | .137 | -.272 | -.201 | .140 |
| Multi-skilled | .036 | .483 | .141 | .082 | -.191 | -.049 | .466 |
| Salary appropriate | .145 | -.359 | .203 | .135 | -.041 | .060 | -.207 |
| Enjoy living in community | -.015 | .032 | .725 | .141 | .046 | -.099 | -.065 |
| Work makes a difference | .016 | -.024 | .723 | -.089 | .019 | .085 | .288 |
| Work valued by community | -.068 | -.007 | .670 | -.090 | .220 | .100 | .084 |
| Get along w colleagues | .095 | .036 | .586 | .215 | -.069 | -.018 | .071 |
| Flexible Hours | .123 | .114 | .020 | .739 | .149 | .121 | -.097 |
| Annual Leave | .052 | -.155 | .011 | .731 | .048 | -.020 | .080 |
| Autonomous | -.054 | -.131 | .291 | .508 | -.105 | .046 | .210 |
| Good facilities | .024 | -.071 | .063 | .121 | .765 | .045 | .007 |
| Good admin support | .186 | -.172 | .083 | .004 | .653 | .191 | -.009 |
| TImely recruitment | -.006 | -.061 | .040 | .030 | .030 | .826 | -.118 |
| Personnel allocation | .140 | -.140 | .034 | -.030 | .108 | .614 | -.109 |
| Manager understands | .283 | -.228 | .025 | .183 | .149 | .469 | .137 |
| CPD Access | .307 | -.095 | -.109 | .157 | .133 | .341 | .169 |
| Wide range of skills | .158 | .106 | .176 | .093 | -.135 | -.115 | .711 |
| Area of expertise | -.050 | -.072 | .088 | .023 | .173 | -.006 | .695 |
| Extraction Method: Principal Component Analysis.  Rotation Method: Varimax with Kaiser Normalization. | | | | | | | |
| a. Rotation converged in 6 iterations. | | | | | | | |

**Rotated Component Matrix - Private Cohort**

| **Rotated Component Matrix^a^** | | | | | | |
| --- | --- | --- | --- | --- | --- | --- |
| **PRIVATE** (N=432) | Component | | | | | |
|  | 1 | 2 | 3 | 4 | 5 | 6 |
| Face to face contact | .811 | .111 | .017 | .103 | -.009 | -.015 |
| Access to experts | .748 | -.008 | -.191 | .039 | -.033 | .072 |
| Professional isolation | -.735 | -.097 | .293 | -.119 | -.058 | -.004 |
| Good clinical support | .629 | .126 | -.040 | .110 | .430 | -.115 |
| CPD Access | .487 | -.158 | -.125 | -.034 | .285 | .152 |
| Enjoy living in community | .108 | .771 | .069 | .103 | -.008 | .076 |
| Work valued by community | .021 | .741 | .080 | .252 | .079 | .023 |
| Work makes a difference | .026 | .672 | .043 | .088 | .149 | .328 |
| Get along w colleagues | -.017 | .550 | -.192 | -.161 | .176 | .116 |
| Beyond boundaries | -.027 | -.028 | .739 | .034 | -.011 | .002 |
| Gaps - limited HR | -.279 | .036 | .657 | -.040 | -.109 | .000 |
| Burned out | -.194 | -.118 | .527 | -.403 | .113 | -.068 |
| Salary appropriate | .130 | -.123 | -.392 | .207 | .237 | .048 |
| Flexible Hours | .021 | .184 | .029 | .810 | .086 | -.028 |
| Annual Leave | .141 | .059 | -.078 | .747 | .045 | -.002 |
| Reasonable workload | .161 | -.070 | *-.421* | .470 | .122 | .033 |
| Good admin support | .092 | .186 | -.098 | .123 | .794 | .015 |
| Good facilities | .119 | .135 | -.034 | .058 | .773 | .160 |
| Wide range of skills | .201 | .289 | .025 | -.137 | -.047 | .701 |
| Multi-skilled | -.041 | .004 | .483 | .068 | -.004 | .644 |
| Area of expertise | -.011 | .166 | -.212 | .050 | .174 | .554 |
| Autonomous | -.135 | .081 | -.198 | .445 | .274 | .452 |
| Extraction Method: Principal Component Analysis.  Rotation Method: Varimax with Kaiser Normalization. | | | | | | |
| a. Rotation converged in 7 iterations. | | | | | | |

**Correlation matrix – Public cohort**

| **PUBLIC cohort** | | F1 Professional Isolation (r) | F2 Sense of Community | F3 Clinical Demand | F4 Getting Time Away | F5 Resources | F7 Management (Public) |  |
| --- | --- | --- | --- | --- | --- | --- | --- | --- |
| F1 Professional Isolation (r) | Correlation | 1 | .052 | .307^**^ | .184^**^ | .324^**^ | .379^**^ |  |
|  | Sig. |  | .147 | .000 | .000 | .000 | .000 |  |
|  | N | 792 | 770 | 777 | 763 | 783 | 720 |  |
| F2 Sense of Community | Correlation | .052 | 1 | .047 | .197^**^ | .141^**^ | .067 |  |
|  | Sig. | .147 |  | .185 | .000 | .000 | .069 |  |
|  | N | 770 | 804 | 782 | 774 | 789 | 727 |  |
| F3 Clinical Demand | Correlation | .307^**^ | .047 | 1 | .197^**^ | .323^**^ | .396^**^ |  |
|  | Sig. | .000 | .185 |  | .000 | .000 | .000 |  |
|  | N | 777 | 782 | 806 | 777 | 794 | 732 |  |
| F4 Getting Time Away | Correlation | .184^**^ | .197^**^ | .197^**^ | 1 | .126^**^ | .167^**^ |  |
|  | Sig. | .000 | .000 | .000 |  | .000 | .000 |  |
|  | N | 763 | 774 | 777 | 798 | 782 | 722 |  |
| F5 Resources | Correlation | .324^**^ | .141^**^ | .323^**^ | .126^**^ | 1 | .328^**^ |  |
|  | Sig. | .000 | .000 | .000 | .000 |  | .000 |  |
|  | N | 783 | 789 | 794 | 782 | 813 | 737 |  |
| F7 Management (Public) | Correlation | .379^**^ | .067 | .396^**^ | .167^**^ | .328^**^ | 1 |  |
|  | Sig. | .000 | .069 | .000 | .000 | .000 |  |  |
|  | N | 720 | 727 | 732 | 722 | 737 | 746 |  |
| **. Pearson Correlation is significant at the 0.01 level (2-tailed). | | | | | | | | |
| *. Pearson Correlation is significant at the 0.05 level (2-tailed). | | | | | | | | |

**Correlation matrix – Private cohort**

| **PRIVATE cohort** | | F1 Professional Isolation (r) | F2 Sense of Community | F3 Clinical Demand | F4 Getting Time Away | F5 Resources | F7 Management (Public) |
| --- | --- | --- | --- | --- | --- | --- | --- |
| F1 Professional Isolation (r) | Correlation | 1 | .122^**^ | .413^**^ | .139^**^ | .297^**^ | .462^**^ |
|  | Sig. |  | .004 | .000 | .001 | .000 | .000 |
|  | N | 629 | 575 | 522 | 597 | 590 | 290 |
| F2 Sense of Community | Correlation | .122^**^ | 1 | .058 | .269^**^ | .306^**^ | .141^*^ |
|  | Sig. | .004 |  | .187 | .000 | .000 | .015 |
|  | N | 575 | 654 | 523 | 609 | 613 | 294 |
| F3 Clinical Demand | Correlation | .413^**^ | .058 | 1 | .344^**^ | .259^**^ | .475^**^ |
|  | Sig. | .000 | .187 |  | .000 | .000 | .000 |
|  | N | 522 | 523 | 577 | 551 | 540 | 290 |
| F4 Getting Time Away | Correlation | .139^**^ | .269^**^ | .344^**^ | 1 | .268^**^ | .321^**^ |
|  | Sig. | .001 | .000 | .000 |  | .000 | .000 |
|  | N | 597 | 609 | 551 | 704 | 635 | 287 |
| F5 Resources | Correlation | .297^**^ | .306^**^ | .259^**^ | .268^**^ | 1 | .422^**^ |
|  | Sig. | .000 | .000 | .000 | .000 |  | .000 |
|  | N | 590 | 613 | 540 | 635 | 676 | 296 |
| **. Pearson Correlation is significant at the 0.01 level (2-tailed). | | | | | | | |
| *. Pearson Correlation is significant at the 0.05 level (2-tailed). | | | | | | | |
